# Online cognitive behavioral group therapy (iCBT-I) for insomnia for school children and their parents: Adaptation of an established treatment (KiSS training)

**DOI:** 10.1007/s11818-020-00280-7

**Published:** 2020-11-09

**Authors:** Angelika A. Schlarb, Hannah Schulte, Anika Selbmann, Ina Och

**Affiliations:** 1 Department of Psychology and Sports Science, Bielefeld University, Universitätsstraße 25, 33615 Bielefeld, Germany; 2 HaKiJu Hochschulambulanz für Kinder und Jugendliche und ihre Familien, Bielefeld University, Morgenbreede 12, 33615 Bielefeld, Germany

**Keywords:** SARS-CoV‑2, COVID 19, Sleep problems, Sleep treatment, Nightmares, SARS-CoV-2, COVID-19, Schlafprobleme, Schlafbehandlung, Alpträume

## Abstract

**Background:**

Due to the SARS-CoV‑2 crisis, online adaptation of sleep trainings is necessary. As sleep disturbances in school children are common, prevention of chronification is essential. The aim of this study was to adapt an established age-oriented cognitive behavioral therapy for insomnia (CBT-I) group training for 5–10-year-old children with insomnia and their parents to an online version (group iCBT-I).

**Methods:**

The adaptation procedure and structure of the iCBT‑I are described. To assess acceptance the Online Sleep Treatment Acceptance questionnaire (OSTA) and the Online Sleep Treatment Feedback questionnaire (OSTF) were implemented. In addition, trainers filled in the Adherence and Feasibility Questionnaire for Online Sleep Treatment (AFOST). Sleep problems were assessed using a structured interview for sleep disorders in children and clinical interview, and the Children’s Sleep Habit Questionnaire (CSHQ-DE). Emotional problems were evaluated with the Child Behavior Checklist (CBCL 4-18).

**Results:**

This pilot study included 12 parents and 6 children fulfilling insomnia criteria prior to online training. The adapted online version consisted of three parental sessions, whereas child-oriented sessions were transferred into videoclips. The new group iCBT‑I was well accepted by parents. Parents scored the online version as helpful and time saving based on the OSTA and trainers estimated the adapted version to be feasible and effective. According to AFOST, adherence was given. After training, 67% of children showed reduced sleep problems according to parental rating.

**Conclusion:**

Parental acceptance of a group iCBT‑I for school children and their parents was very good and parents scored the videos for their children as very helpful. Trainers declared the adapted version to be feasible. A further study with a larger sample is necessary.

## Abstract (de)

**Hintergrund:**

Wegen der SARS-CoV-2-Krise ist die Online-Adaptation von Schlaftrainings notwendig. Da Schlafstörungen bei Schulkindern häufig sind, ist die Prävention der Chronifizierung essenziell. Ziel der vorliegenden Studie war es, ein etabliertes altersorientiertes Gruppentraining mit kognitiver Verhaltenstherapie bei Schlafstörungen (CBT-I) für 5‑ bis 10-jährige Schulkinder mit Schlafstörungen und deren Eltern als Online-Version zu adaptieren (Gruppen-iCBT-I).

**Methoden:**

Der Adaptationsprozess und die Struktur des iCBT‑I werden beschrieben. Zur Erfassung der Akzeptanz wurden die Fragebögen Online Sleep Treatment Acceptance (OSTA) und Online Sleep Treatment Feedback (OSTF) eingesetzt. Zusätzlich füllten die Trainer den Adherence and Feasibility Questionnaire for Online Sleep Treatment (AFOST) aus. Schlafprobleme wurden anhand eines strukturierten Interviews für Schlafstörungen bei Kindern und eines klinischen Gesprächs sowie des Children’s Sleep Habit Questionnaire (CSHQ-DE) erfragt. Emotionale Probleme wurden mit der Child Behavior Checklist (CBCL 4-18) beurteilt.

**Ergebnisse:**

Diese Pilotstudie erfolgte mit 12 Elternteilen und 6 Kindern, welche vor dem Online-Training die Kriterien einer Schlafstörung erfüllten. Die adaptierte Online-Version bestand aus 3 Elternsitzungen, während die kindorientierten Sitzungen in Videoclips übertragen wurden. Das neue Gruppen-iCBT‑I wurde von den Eltern gut angenommen. Die Eltern beurteilten auf Basis des OSTA die Online-Version als hilfreich und zeitsparend, und die Trainer schätzten die adaptierte Version als brauchbar und effektiv ein. Gemäß AFOST war die Adhärenz gegeben. Der elterlichen Beurteilung zufolge wiesen nach dem Training wiesen 67% der Kinder verminderte Schlafprobleme auf.

**Schlussfolgerung:**

Die elterliche Akzeptanz eines Gruppen-iCBT‑I für Schulkinder und ihre Eltern war sehr gut, und die Eltern beurteilten die Videos für ihre Kinder als sehr hilfreich. Die Trainer hielten den Einsatz der adaptierten Version für praktikabel. Nun ist noch eine weitere Studie mit einer größeren Stichprobe notwendig.

## Background

Sleep initiation and maintenance disorders are common among school children. Such sleep problems in children can cause mental and physical health impairments, including susceptibility to infections and other medical issues.

The severe acute respiratory syndrome coronavirus 2 (SARS-CoV-2) outbreak beginning 2019/2020 has become a global pandemic. In order to reduce disease spread, citizens of all countries were required to stay at home (lockdown). For children and families this was associated with extended kindergarten and school closures. The side effects of lockdown were hypothesized to be extensive and include sleep problems, heightened stress, anxiety, and other outcomes. Dellagiulia et al. [5] examined the impact on children’s sleep quality. They evaluated inter- and intraindividual daily variation of preschooler’s sleep duration and quality over 4 weeks. They found that at the beginning of lockdown, parents reported more challenging bedtime routines and a decrease in children’s sleep quality. Subsequently, sleep routine and sleep quality stabilized at a lower level. Furthermore, sleep duration decreased and then stabilized as well. However, sufficient and healthy sleep of high quality is important for the immune system [4]. Therefore, sleep interventions to support family wellbeing should be implemented from early on—especially during a pandemic situation such as COVID-19. To support health care services during this critical phase, a task force of the European Academy for Cognitive Behavioral Therapy for Insomnia (CBT-I) formulated practical sleep-related recommendations [3]. The efficiency of cognitive CBT‑I for infants and young children with sleep problems has been tested in various studies [11, 12, 17]. However, data on age-oriented CBT‑I treatment of sleep problems in children is still sparse and the number of studies concerning CBT‑I for children is quite low [6, 9]. In the authors’ previous work, they evaluated the long-term effectiveness of a CBT‑I program in groups for children with insomnia aged 5–10 years. The acceptance and efficiency was assessed in an early pilot study of the KiSS (*Training for Kinder mit Schlaf-Störungen*; [20]) as were long-term effects of this CBT‑I group treatment containing CBT‑I and imaginary or hypnotherapeutic elements [16]. However, there are only sparse possibilities for treating sleep-disordered children without face-to-face interaction. A review found only six studies concerning web-based treatments for children and their parents in the form of a multicomponent intervention. In these studies, interventions for children were parent focused. However, telehealth interventions may represent a future direction to reduce barriers and problems of delivery regarding adequate treatments for sleep problems in children. Due to the SARS-CoV‑2 crisis, new treatment approaches based on evaluated studies are necessary to help from early on. To the best of the authors’ knowledge, there is no evaluated online group treatment available that provides scientifically based and age-oriented information and behavioral strategies for parents and children. Therefore, the overall objective of the current study was to develop and examine the acceptance, adherence, and efficacy of an iCBT‑I group treatment for sleep disturbances for children aged 5–10 years and their parents. Based on previous studies concerning KiSS, the authors wanted to adapt the CBT‑I group training to an internet-based CBT‑I (iCBT-I) group treatment. The adaptation procedure and acceptance are described precisely and the first effects evaluated exploratively.

The study hypotheses were (1) that the treatment would be well accepted by parents, (2) that the video elements would be age oriented, (3) that the treatment would be well accepted by trainers, and (4) that the treatment would improve the sleep and mental health of children.

## Methods

### Materials and methods

#### Participants

Inclusion criteria were age of the child between 5 and 10 years and insomnia; 12 parents with 6 children participated in this pilot study. The age of the parents ranged from 40 to 51 years (mothers 40–44 years, fathers 40–51 years). Children were aged 5–10 years (youngest 5.4 years, oldest 10.10 years), with two girls and four boys. All children fulfilled insomnia criteria based on the International Classification of Diseases (ICD)-10 and the Diagnostic and Statistical Manual of Mental Disorders (DSM)-5 as well as International Classification of Sleep Disorders (ICSD‑3). Two children also had nightmares. Daytime impairments were also reported for three children who suffered from daytime tiredness. Parents of all children reported significant sleep problems of their child.

The training was held in online group conditions with 2–3 parents participating per group. Diagnostic procedures and treatment were conducted according to standard ethical guidelines as defined by the Declaration of Helsinki and the KiSS study was approved by the ethics committee of Bielefeld University.

### Measurements

#### Structured interview for sleep disorders in children and clinical interview

A structured parent interview for the diagnosis of sleep disorders in children aged 5–10 years was conducted during the initial consultation. The diagnostic staff (psychologists, child and adolescent psychotherapists) had been previously trained. All interviews were conducted according to this standardized procedure. The interview describes all sleep disorder diagnoses and isolated symptoms of the International Classification of Sleep Disorders (ICSD)-3, DSM‑5, and ICD-10 relevant for sleep disorders in children at this age. The answers were scored by the interviewer using the following four alternatives: “?” (no or insufficient information); “1” (“criterion is not met”); “2” (“criterion not fully met”); and “3” (“criterion fully met”). The duration of each interview varied between 30 and 50 min.

#### Children’s Sleep Habits Questionnaire

The children’s sleep behavior was assessed in the form of parental report using the German version of the Children’s Sleep Habits Questionnaire (CSHQ) [13], CSHQ-DE [19]. With eight subscales (sleep anxiety, bedtime resistance, night waking, sleep duration, sleep onset delay [SOD], daytime sleepiness, sleep-disordered breathing, parasomnias) and a total sleep disturbance score (TSDS), both behavioral and medically based sleep problems in school-aged children are identified. Internal consistency for a clinical sample (*p* = 0.78) and test–retest reliability (range 0.62–0.79) were acceptable. A value of 41 was determined as the most sensitive and specific cut-off score. CSHQ-DE has also been validated and its psychometric properties shown to be adequate [19].

#### Child Behavior Checklist

To determine behavioral and emotional problems in children, parents completed the Child Behavior Checklist (CBCL 4–18, [2]). Eight clinical subscales (withdrawal, somatic complaints, anxious/depressed, social problems, thought problems, attention problems, delinquent behavior, and aggressive behavior) are summarized to scores for internalizing and externalizing problems as well as a total score. Test–retest reliability was 0.89 for internalizing and 0.93 for externalizing problems [1]. The clinical cut-off for t‑values is defined as t ≥ 64, while values between t = 60 and t = 63 are determined as boundary area.

#### Online Sleep Treatment Acceptance Questionnaire

To evaluate the acceptance of all contents of the online group KiSS treatment, a specific questionnaire, the Online Sleep Treatment Acceptance Questionnaire (OSTA), was constructed according to Schlarb and Brandhorst [17]. The questionnaire were constructed with regard to the following areas: first, to assess the acceptance of the contents of the treatment; second, to provide an overall evaluation of the training; finally, they had space for remarks and recommendations concerning the training. The questionnaire contained 11 questions. Parents rated on a five-point Likert scale (1 = I fully agree, 2 = I rather agree, 3 = I don’t know, 4 = I rather disagree, 5 = I fully disagree).

#### Online Sleep Treatment Feedback Questionnaire

Beyond the OSTA questionnaire, parents were also asked to complete the Online Sleep Treatment Feedback Questionnaire (OSTF). This questionnaire contained eight questions regarding the technical aspects of the online treatment. Questions included parental and child perspectives (e.g., “Our child implemented the learned strategies of the videos with enjoyment”). Parents rated the items on a five-point Likert scale (1 = I fully agree, 2 = I rather agree, 3 = I don’t know, 4 = I rather disagree, 5 = I fully disagree).

#### Adherence and Feasibility Questionnaire for Online Sleep Treatment

Trainers filled in the Adherence and Feasibility Questionnaire for Online Sleep Treatment (AFOST) concerning the online adaptation of the KiSS group treatment. Next to adherence and feasibility, they were asked about parental acceptance and feasibility. In addition, several questions addressed technical issues. The questionnaire contained 18 items in total. An additional field addressed recommendations regarding future developments. They rated the items on a five-point Likert scale (1 = I fully agree, 2 = I rather agree, 3 = I don’t know, 4 = I rather disagree, 5 = I fully disagree).

### Procedure

Before participation, families received an information letter and gave their written informed consent. Furthermore, inclusion criteria (age of the child, insomnia symptoms according to the international classification of sleep disorders ICSD‑3, DSM‑5, ICD-10) were checked. In addition, parents completed a sleep and medical history questionnaire for further screening for eligibility criteria. To exclude somatic causes of the sleep problem and medical conditions, each family had to consult their pediatrician before taking part in the study. Insomnia was defined as trouble falling asleep (operationalization: ≥30 min; ≥ five times per week; ≥ three consecutive months) or trouble sleeping through the night (operationalization: waking up ≥ once per night and trouble falling asleep again; ≥ five times per week; ≥ three consecutive months). Exclusion criteria were somatic/medical reasons for sleep disorder (confirmed by pediatrician before study participation) and child in any psychotherapeutic/mental health treatment.

### Online group intervention adaptation—iCBT-I for children

The original CBT‑I program KiSS was created for children aged 5–10 years suffering from sleep disorders (insomnia and nightmares) and their parents. Contents of the group CBT‑I were formalized in a manual [15]. For a detailed description of the treatment components, see Schlarb et al. [16], Schlarb and Bihlmaier [15]. Adaptation of parental sessions was relatively easy as the original manual gives detailed advice concerning parental behavior strategies for coping with the sleep problems of their child. However, all strategies had to be adapted to the online treatment modality (advices, homework, etc.). In addition, the group size was restricted to only 2–3 families, as the doccura software (Bayerische Telemed Allianz GmbH; Baar-Ebenhausen) allowed no larger group sizes. In addition, parents were instructed regarding the homework concerning parental strategies. All homework was additionally manualized and explained in detail in child-oriented strategies and videos.

The most difficult part was the adaptation of the original children’s sessions (three) with “Kalimba” as a coping model and sleep expert, because all strategies are implemented and strengthened by the sleep therapy puppet Kalimba, a leopard with magical power spots for good sleep. In addition, Kalimba encourages the child to implement newly learned sleep-related strategies. Moreover, Kalimba serves as assistance helping children to gain competences of sleeping without their parents in an appropriate child-friendly way (for details see [20]). The original training uses lots of role playing between Kalimba and the trainer (the interviewer of Kalimba); therefore, the adaptation was rather tricky. However, we transformed the role plays into a video format with Kalimba and a child puppet asking the questions about sleep and sleep-related intervention strategies. Parents were instructed on how to be the trainer (“train to train,” which is also a part of the original manual) and viewed and discussed the sleep videos with their children. More detailed information regarding intervention videos is provided in Table 1.

**Table 1:** Contents and duration of children’s videoclips

Session	Video contents	Duration of video
Child session 1	Video 1: introduction to Kalimba, greeting of the children, explaining the magical power of Kalimba, sleep problem history of Kalimba (coping model), magical spots of Kalimba, sleeping map, introduction of the reward system	5:58
	Video 2: repetition of learned strategies, magic world of Kalimba, story of a child with sleep problems, how to use the magic spots to sleep well, how to do magical sleep spells with Kalimba	8:41
	Video 3: repetition of learned strategies, how to use the sleep stars during the night, relaxation strategies with Kalimba, homework	7:03
Child session 2	Video 1: repetition of learned strategies, discussion of the homework, further magic spots of Kalimba (sleep onset problems), becoming relaxed with a magic spot, breathing technique, having nice dreams, strategies against nightmares	5:07
	Video 2: repetition of learned strategies, sleeping environment, how to create a nice bed and sleeping environment (bedsheets, quietness, etc.), worries and the sorrow box	9:37
	Video 3: repetition of learned strategies, how to fight against nightmares with Kalimba, Kalimba and his friends, painting strategies against nightmares, how to become a sleep hero, my sleep hero story	5:56
Child session 3	Video 1: repetition of learned strategies, sorrow box, painting nightmares, sleep hero story, sorrow box, breathing technique, working further with Kalimba (using the other spots), sleep stars, learned strategies and tricks, my sleeping box, Kalimba says bye bye	5:08

### Analysis

As data were assessed regarding acceptance, feasibility, and adherence of online group treatment, data analysis was conducted descriptively. In addition, data of 12 parents and 6 children regarding training and sleep and emotional health were assessed. Therefore, data are presented as a pilot study. Although, the sample of this pilot study was quite small, it follows Fricke and Lehmkuhl [7], who also published only single-case descriptive data as a pilot study.

## Results

From the *N* = 12 parents with 6 children participating in the iCBT‑I KiSS training, feedback data regarding online treatment were available from all parents. In addition, parents of four children returned the CSHQ and the CBCL 4-18.

The core concern was to evaluate the acceptance of online KiSS in an iCBT‑I group format from the perspective of parents and trainers. Therefore, the following sections firstly describe the results of OSTA and OSTF regarding acceptance of online KiSS. In addition, the trainers’ feedback forms concerning adherence and feasibility are presented. Concerning sleep and emotional health, pre- and postmeasurement data of families who returned the postmeasurement questionnaires (*n* = 4) are presented. Therefore, the results of the pilot study are presented in a descriptive manner.

### Acceptance

To measure acceptance, parents rated all topics of online KiSS with the OSTA on a five-point Likert scale. In sum, more than 80% of parents rated most modules as adequate, 74.9% found tips and tricks concerning sleep problem-solving helpful, and 91% found the group format very helpful. More than 50% rated the online format as comfortable.

Videos were rated by 75% of parents as helpful for them as parents. Further detailed feedback is presented in Table 2.

**Table 2:** Results of the OSTA (*N* = 11 parents completed the questionnaire)

	I fully disagree 5	I rather disagree 4	I don’t know 3	I rather agree 2	I fully agree 1
The topics of the sessions were important	–	–	16.7%	41.7%	33.3%
Theoretical and practical knowledge was presented in an easy way	–	–	–	58.3%	33.3%
Our family situation and circumstances were well considered	–	–	–	58.3%	33.3%
I got answers to my questions	–	–	8.3%	41.7%	41.7%
I received recommendations for daily routine	–	–	8.3%	8.3%	75%
We had practical exercises	–	–	16.7%	41.7%	33.3%
I received concrete tips, help, and advice for home training	–	–	16.7%	41.7%	33.3%
I felt comfortable in the group	–	–	16.7%	58.3%	16.7%
We had enough time to exchange experiences	–	–	–	83.3%	8.3%
This session motivated me to work on our sleep problem	–	–	8.3%	41.7%	41.7%
I am fond of the online version of the sleep training	8.3%	–	8.3%	50%	–

Number of answers vary due to missing data

*OSTA *Online Sleep Treatment Acceptance questionnaire

### Qualitative feedback (open-answer format)

Parents also filled in the qualitative field of the questionnaire. The following remarks were given:

“My child was very fond of the videos and Kalimba was very well accepted,” “The discussion was very helpful despite the video format,” “Although there were technical burdens, the online format was very good,” “As the training was online, I saved some time,” “My child was able to learn very efficiently with the videos and could transfer strategies into daytime practice.”

In addition to the contents of the training, the implemented videos were also evaluated in detail. Most of the parents and their children liked the videos. They scored them as helpful for teaching their children the sleep strategies. However, while one third did not use the consultation hours to ask questions, more than 90% rated the materials as useful. Detailed results regarding the OSTF are presented in Table 3.

**Table 3:** Parental reports of video interventions (*N* = 12)

	I fully disagree 5	I rather disagree 4	I don’t know 3	I rather agree 2	I fully agree 1
We (parents) liked the videos	–	–	25%	58.1%	16.7%
My child liked the videos	–	–	25%	50%	25%
The videos were helpful for us (parents) to teach our child and implement the strategies into daily routine	–	–	–	75%	25%
The videos helped our child to practice the recommended strategies	–	–	33.3%	50%	16.7%
Our child liked training the strategies in the videos	–	–	16.7%	75%	8.3%
Instructions for us (parents) were clear and understandable so that we could train our child easily	–	–	25%	75%	–
For questions regarding videos and materials we used the virtual KiSS consultation hours	33.3%	–	–	33.3%	25%
The materials were useful for training our children	–	–	8.3%	91.7%	–

Number of answers vary due to missing data

### Adherence and acceptance of trainers

Regarding the results of the Adherence and Feasibility Questionnaire for Online Sleep Treatment (AFOST), a total of *N* = 2 trainers conducted the iCBT‑I trainings. Each of them completed the AFOST anonymously. Overall, trainers rated the elements of the online adaptation as effective and were able to conduct the training in the groups. Besides, both felt comfortable in the groups. However, more time for group discussion was recommended in addition to an esthetical adaptation of the videos (camera position).

Detailed feedback of the trainers regarding their perspective of families is presented in Tables 4 and 5.

**Table 4:** Trainer 1 ratings

	I fully disagree 5	I rather disagree 4	I don’t know 3	I rather agree 2	I fully agree 1
Parents were fond of the topics	–	–	–	–	X
We could teach theoretical and practical knowledge in an understandable way	–	–	–	–	X
Parents could understand theoretical and practical knowledge	–	–	–	X	–
We could address the families individually	–	–	–	–	X
Parents reported that we addressed their personal situation	–	–	–	–	X
We were able to answer their questions	–	–	–	–	X
We could give advice and recommendations	–	–	–	–	X
Parents trained the strategies	–	–	–	X	–
Parents watched the videos with their children and discussed them	–	–	–	X	–
We could give concrete advice for transfer into daily routine	–	–	–	–	X
I felt comfortable in the groups	–	–	–	–	X
They had enough time to discuss and exchange experiences	–	–	–	–	X
The parents were motivated to work on the sleep problem after the training	–	–	–	X	–
The videos were easy to conduct	–	–	–	X	–
The online version was easy to implement	–	–	–	X	–
We could easily follow the manual (adherence)	–	–	–	–	X
The training was easy to conduct	–	–	–	–	X
The videos were age oriented	–	–	–	–	X

**Table 5:** Trainer 2 ratings

	I fully disagree 5	I rather disagree 4	I don’t know 3	I rather agree 2	I fully agree 1
Parents were fond of the topics	–	–	–	X	–
We could teach theoretical and practical knowledge in an understandable way	–	–	–	–	X
Parents could understand theoretical and practical knowledge	–	–	–	X	–
We could address the families individually	–	–	–	–	X
Parents reported that we addressed their personal situation	–	–	–	–	X
We were able to answer their questions	–	–	–	–	X
We could give advice and recommendations	–	–	–	–	X
Parents trained the strategies	–	–	–	X	–
Parents watched the videos with their children and discussed them	–	–	–	X	–
We could give concrete advice for transfer into daily routine	–	–	–	X	–
I felt comfortable in the groups	–	–	–	–	X
They had enough time to discuss and exchange experiences	–	–	–	–	X
The parents were motivated to work on the sleep problem after the training	–	–	–	X	–
The videos were easy to conduct	–	–	X	–	–
The online version was easy to implement	–	–	–	X	–
We could easily follow the manual (adherence)	–	–	–	–	X
The training was easy to conduct	–	–	–	X	–
The videos were age oriented	–	–	–	–	X

#### Recommendations for improvements (open-answer format)

Trainer suggestions for improvement were the following: “more time for discussion in the groups,” “videos could be nicer with more precise image sections,” “more time at the end of the sessions to instruct parents as co-trainers.”

### First effects

Besides feasibility, acceptance, and adherence, the sleep-related effect was also evaluated in this pilot study. Therefore, sleep-related feedback was assessed by trainers and sleep changes were scored on the CSHQ and via sleep items of the CBCL. In addition, mental health based on the CBCL is reported, pre–post data are presented:

All in all, 67% of the children slept better after the training according to parental report based on clinical interview. Insomnia severity decreased to a nonclinical level. The mean score in the CSHQ decreased from mean (M) = 51.6 to M = 44.6 after online training. Mental health based on the CBCL was at M = 61 prior to online treatment and M = 58.3 after training.

## Discussion

As new approaches to sleep help are required—especially due to the corona crisis—an online version of an established child-oriented group CBT‑I would be beneficial. Therefore, the main goal of this study was to develop an online treatment for parents of children aged 5 to 10 years suffering from insomnia. First of all, the authors wanted to explore the acceptance and feasibility of such a treatment from the parental perspective. In addition, the experiences and adherence of the trainers was also to be explored. Besides these main topics, sleep-related effects were also assessed based on interview data, questionnaires, and mental health. The current study adapted a well-established group sleep treatment (KiSS) for children and their parents to a group iCBT‑I treatment. Furthermore, the authors wanted to reduce barriers for various families to enter sleep intervention and help to prevent chronification. Overall, the adapted version of the KiSS treatment—also in a group format—was well accepted by the families. Most scored the treatment as helpful regarding the child’s sleep problems.

The results of this study indicate that the internet-adapted group intervention of KiSS is very well accepted by parents and trainers. Most parents would recommend the treatment to other families, reported being able to deal with their children’s sleep-related problems after training, and scored the videos as helpful and age oriented. These results are in line with the Mini-KiSS online treatment addressing parents of younger children [17]. Furthermore, the results showed that the treatment succeeded in improving sleep problems according to parents and trainers. However, the CSHQ scores were still above the cut-off of 41 directly after training.

The online KiSS intervention addressed not only parents with parental behavior strategies but also provided information about children’s sleep and train-to-train recommendations. In online sessions, parents were instructed to identify difficult sleep-related situations and received parental behavior recommendations concerning their child’s sleep problem (like Mini-KiSS), but online KiSS group training also implemented child-oriented elements due to the videos. In addition, effective child-oriented therapeutic strategies such as role play, coping model, and implementation of reward systems could be adapted in online KiSS. However, in contrast to the evaluated KiSS treatment [16], no children sessions were included. Child-oriented parts were delegated to the parents. Furthermore, in contrast to another study [10], all parents participated in the same structured online group intervention without any additional or varied treatment conditions.

According to more than 90% of the parents, the materials were helpful for changing their child’s sleep problems. In addition, all rated the videos as helpful for changing the sleep problem. These results are in line with others showing that the Internet is often used as a source of sleep-related information. First hints were found that parents of children are able to function as cotrainers to instruct their children and help them to sleep better with age-oriented supportive materials and specific parental training. In contrast to other studies, the study at hand was an online group intervention. Most of the previous published studies focused on younger children and/or were based on the individual treatment condition and not in groups [10, 17]. Beyond this, the current findings widen the results concerning other age groups such as adults demonstrating the efficacy of an Internet-based intervention [14, 21, 22].

As a specific age group (school children) showing a high prevalence of sleep problems [18] and their parents were addressed, early intervention and prevention of chronification is very important. As some families reported, the online intervention was time saving, as they did not need to come to the outpatient clinic for treatment. In addition, such an intervention format might be especially helpful for families with more than one child, as they do not need to find a babysitter for the sessions, and for special families (e.g., single parents). Future research should keep this in mind.

Concerning treatment application, in contrast to a previous study addressing younger children, the present study had some kind of personal contact (via video consultation) but without meetings in person [17]. Therefore, unspecific treatment effects such as those discussed by Grawe were more active than in a purely online version without any trainer contact [8].

### Limitations

There are some limitations of the study that should be kept in mind. First, only 12 parents with 6 children participated in this pilot study addressing the adaptation and feasibility of an online format using videos for children. Therefore, the results have to be considered with caution. However, detailed feedback was received from parents regarding the group training format, the elements for the children, and technical issues. In addition, we also evaluated the trainers’ experiences concerning the various facets of the training. However, future studies based on such an online format should integrate further and more detailed sleep measures such as actigraphy and also parental cognitions regarding their child’s sleep. This pilot study also included no follow-up measurements. Future studies implementing long-term outcomes are required. However, the focus and interest of the study at hand was to adapt the manual KiSS group intervention to an online format, assess the acceptance and feasibility of the adapted version in parents with children suffering from insomnia, and further assess the trainer evaluations.

## Conclusion

The study at hand addressed the online adaptation of a group CBT‑I training (KiSS) and therefore reacted to requirements for new approaches to treatment brought about by the SARS-CoV‑2 crisis, as recommended by the European CBT‑I Academy [3]. This pilot study shows that the online group format is possible for parents of schoolchildren and, further, that child-oriented treatment elements can be transferred into videoclips.

## References

[1] Achenbach TM (1991). Integrative guide for the 1991 CBCL/4-18, YSR and TRF profiles.

[2] Achenbach TM, Edelbrock CS (1983). Manual for the childbehavior checklist and revised behavior profile.

[3] Altena E, Baglioni C, Espie CA, Ellis J, Gavriloff D, Holzinger B, Schlarb A, Frase L, Jernelöv S, Riemann D (2020). Dealing with sleep problems during home confinement due to the COVID-19 outbreak: practical recommendations from a task force of the European CBT-I academy. J Sleep Res.

[4] Besedovsky L, Lange T, Haack M (2019). The sleep-immune crosstalk in health and disease. Physiol Rev.

[5] Dellagiulia A, Lionetti F, Fasolo M, Verderame C, Sperati A, Alessandri G (2020). Early impact of COVID-19 lockdown on children’s sleep: a 4-week longitudinal study. J Clin Sleep Med.

[6] Dewald-Kaufmann J, de Bruin E, Michael G (2019). Cognitive behavioral therapy for insomnia (CBT-i) in school-aged children and adolescents. Sleep Med Clin.

[7] Fricke L, Lehmkuhl G (2006). Schlafstörungen im Kindes- und Jugendalter: Ein Therapiemanual für die Praxis.

[8] Grawe K (2004). Psychological Therapy.

[9] Meltzer LJ, Mindell JA (2014). Systematic review and meta-analysis of behavioral interventions for pediatric insomnia. J Pediatr Psychol.

[10] Mindell JA, Du Mond CE, Sadeh A, Telofski LS, Kulkarni N, Gunn E (2011). Efficacy of an Internet-based intervention for infant and toddler sleep disturbances. Sleep.

[11] Mindell JA, Kuhn B, Lewin DS, Meltzer LJ, Sadeh A (2006). Behavioral treatment of bedtime problems and night wakings in infants and young children. Sleep.

[12] Morgenthaler TI, Owens J, Alessi C, Boehlecke B, Brown TM, Coleman J (2006). Practice parameters for behavioral treatment of bedtime problems and night wakings in infants and young children. Sleep.

[13] Owens JA, Spirito A, McGuinn M (2000). The Children’s Sleep Habits Questionnaire (CSHQ): psychometric properties of a survey instrument for school-aged children. Sleep.

[14] Ritterband LM, Thorndike FP, Gonder-Frederick LA, Magee JC, Bailey ET, Saylor DK, Morin CM (2009). Efficacy of an Internet-based behavioral intervention for adults with insomnia. Arch Gen Psychiatry.

[15] Schlarb A, Bihlmaier I (2013). KiSS - Das Schlafstörungsprogramm für Kinder zwischen fünf und zehn Jahren. Schlafmag Wege Gesund Schlaf.

[16] Schlarb AA, Bihlmaier I, Velten-Schurian K, Poets CF, Hautzinger M (2016). Short- and long-term effects of CBT-I in groups for school-age children suffering from chronic insomnia: the KiSS-program. Behav Sleep Med.

[17] Schlarb A, Brandhorst I (2012). Mini-KiSS Online: an Internet-based intervention program for parents of young children with sleep problems—influence on parental behavior and children’s sleep. Nat Sci Sleep.

[18] Schlarb AA, Gulewitsch MD, Hautzinger M (2010). Insomnien in der pädiatrischen Praxis. Somnologie.

[19] Schlarb AA, Schwerdtle B, Hautzinger M (2010). Validation and psychometric properties of the German version of the Children’s Sleep Habits Questionnaire (CSHQ-DE). Somnologie.

[20] Schlarb AA, Velten-Schurian K, Poets CF, Hautzinger M (2011). First effects of a multicomponent treatment for sleep disorders in children. Nat Sci Sleep.

[21] Spiegelhalder K, Acker J, Baumeister H, Büttner-Teleaga A, Danker-Hopfe H, Ebert DD (2020). Digitale Behandlungsangebote für Insomnie – eine Übersichtsarbeit. Somnologie.

[22] Ström L, Pettersson R, Andersson G (2004). Internet-based treatment for insomnia: a controlled evaluation. J Consult Clin Psychol.

